# Pressure stabilization effect on the donor–acceptor polyiodide chains in tetraethylammonium bis(diiodine) triiodide – insights from Raman spectroscopy†

**DOI:** 10.1039/d4dt00268g

**Published:** 2024-02-15

**Authors:** Tomasz Poręba, Piero Macchi, Nicola Casati, Tomasz Sierański

**Affiliations:** a Laboratory for Quantum Magnetism, Institute of Physics, École Polytechnique Federale de Lausanne Lausanne CH-1015 Switzerland tomasz.poreba@esrf.fr; b Department of Chemistry, Materials and Chemical Engineering, Polytechnics of Milan Via Mancinelli 7 20131 Milan Italy; c Swiss Light Source, Paul Scherrer Institute CH-5232 Villigen PSI Switzerland; d Institute of General and Ecological Chemistry, Lodz University of Technology Zeromskiego 116 90-924 Lodz Poland; e European Synchrotron Radiation Facility 71 Avenue des Martyrs 38000 Grenoble France

## Abstract

Polyiodides present high bonding flexibility already at ambient conditions, and undergo significant pressure-induced structural deformations. Resonant Raman spectroscopy has been widely used to study I–I bonds in various polyiodides, but it carries a risk of photodecomposition due to the high visible-light absorption of iodine. In this study, tetraethylammonium (bis)diiodine triiodide (TEAI) has been investigated by resonant Raman spectroscopy up to 12.02(3) GPa. The effect of pressure on the intensities and positions of Raman bands has been evaluated and correlated with the interatomic I–I distances derived from high-pressure X-ray diffraction experiments. Pressure was shown to effectively stabilize TEAI against laser-induced photodecomposition, even after a long course of irradiation with the resonant laser light. Examination of a freshly exposed crystal surface revealed that TEAI superficially passivates with the layer of lower polyiodides, which prevents further iodine loss, and shows distinct pressure-induced behaviour.

## Abstract 2

Polyiodides catenate at high pressure. Compression not only alters their bonding, but also stabilize them against photodecomposition.

## Graphical abstract

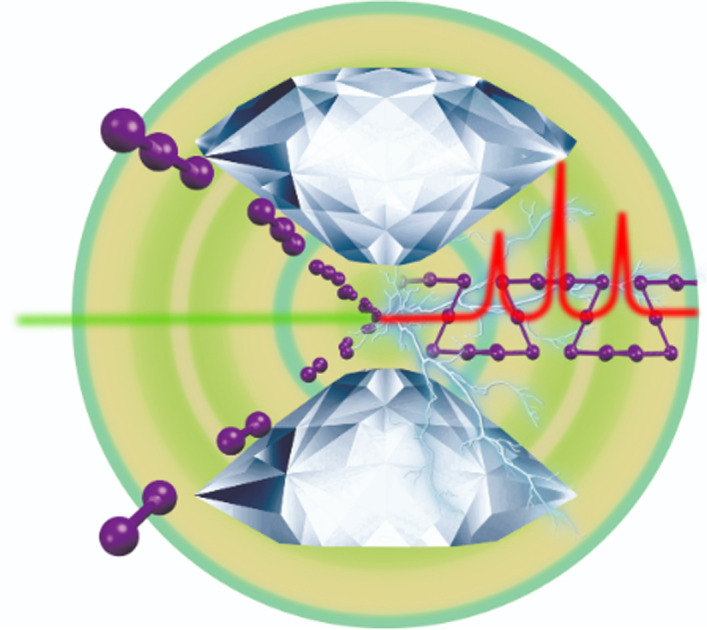

## Introduction

Organic polyiodides (PIs) are utilized in a plethora of practical applications, including molecular conductors, electrolytes, optical devices, antibacterial and antiviral agents, to name a few.^1–5^ The great diversity of their properties in the solid-state often stems from the iodine ability to form complex PI scaffolds, encompassing various sizes and geometries. Raman spectroscopy is predominantly used in the analysis of solid PIs.^6–9^ It aids in identifying the iodine moieties within PIs and neutral iodine adducts and provides a way to evaluate the nature of the I–I interactions. In the case of the simplest polyiodide: symmetric (*D*_∞h_) I_3_^−^, the Raman spectrum is dominated by the fundamental of the *ν*_1_ symmetric stretch at around 112 cm^−1^. In both solutions and in the solid state, the actual symmetry of I_3_^−^ is perturbed by the external electrostatic field, often resulting in activation of a Raman-active asymmetric stretching band (*ν*_2_) around 140 cm^−1^. In fact, only 27% of the I_3_^−^ fragments deposited in the Cambridge Structural Database (CSD) present *D*_∞h_ symmetry.^10^ Numerous asymmetric PIs in the solid state correspond to the relatively low energy barrier required for asymmetrization (*circa* 5 kcal mol^−1^, *vide infra*).^11^ In such asymmetric cases PIs may be better described as intermediates of I_2_ and I^−^ addition reaction, and the peak from the perturbed I_2_ (between 140 and 180 cm^−1^) should be observed, as in I_3_^−^ (I_2_·I^−^), I_5_^−^ (I_2_·I_3_^−^ or 2I_2_·I^−^) *etc*. In the latter case of I_5_^−^, the level of complexity of the Raman spectra increases, as fundamentally three bands are observed: inner asymmetric stretch (around 103 cm^−1^), outer symmetric and asymmetric stretches (around 156 and 141 cm^−1^, respectively).^7^ Therefore, experimentally bands around 100 and 140 cm^−1^ can be either ascribed to I_3_^−^ perturbed by I_2_ or genuine I_5_^−^. Distinguishing between these two situations is often challenging due to the broad and relatively continuous range of I–I bond lengths found in the solid-state structures of PIs.^6^

**Fig. 1:**
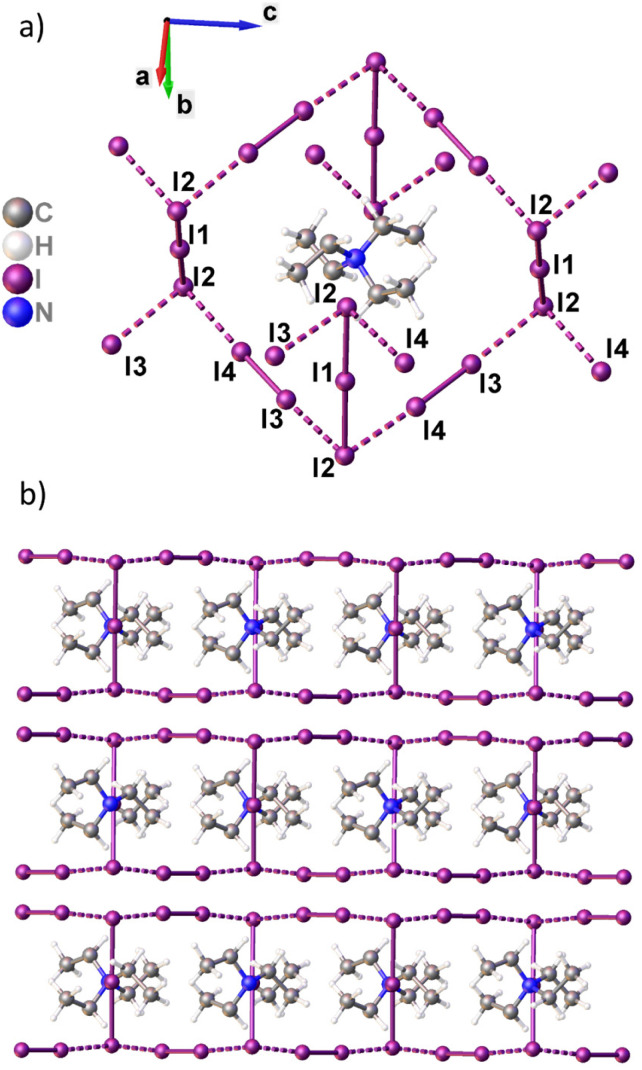
Structure of I_3_^−^⋯I_2_ iodine chains (a) and supramolecular structure of corrugated iodine layers (b) in TEAI.

An additional difficulty in studying PIs with Raman spectroscopy is their susceptibility to laser-induced decomposition. Prolonged irradiation with the visible-light laser can lead to artifacts, and incorrect band assignments.^8^ This decomposition can be attributed to the thermal sublimation of diiodine, a process intensified by high absorption in the visible spectrum, resulting in the disintegration of higher PIs (such as I_5_^−^ or I_8_^2−^), into triiodides or even iodides.^12^ Resonant Raman enhancement has been demonstrated for solid PIs using visible laser excitation sources.^8,13^ Moving from the green to the blue laser, the radiation enters the region of enhanced absorption of the I_3_^−^ species, simultaneously moving away from the absorption maximum for the molecular iodine (Fig. 1). For example, PIs containing I_2_·I_3_^−^ assembly, irradiated with a 514.5 nm laser, resulted in a threefold increase of the intensity ratio *ν*_1_(I_2_)/*ν*_1_(I_3_^−^), compared to a 457.9 nm laser.^13,14^ Utilization of near-infrared lasers was advocated for PIs, since it does not seem to cause iodine loss, fluorescence or sample pyrolysis.^8^ Comparable effects can be obtained with the standard green lasers with the substantial intensity and exposure time reduction, using freshly prepared samples.^15^ Additionally, physical entrapment of iodine within the crystal matrix, such as by applying high pressure (HP), can yield similar outcomes.^16,17^

To date, there are only a few Raman spectroscopy studies on PIs at HP. Investigations into aqueous solutions of KI–I_2_ up to 0.18 GPa revealed no discernible alterations in band frequencies or their relative intensities.^18^ A more recent study on dithiazolylidene–dithiazolium PI revealed a growing association within I_8_^2−^ units above 1.5 GPa.^19^ Iodine-doped poly(vinyl alcohol) films, studied up to 8 GPa, showed enhancement of donor–acceptor interactions within PI chain (I_2_–I^−^–I_2_ fragments), with subsequent breaking into I_3_^−^ and I_2_.^20^ Pressure of 7 GPa was used to locate and characterize the nature of I_*n*_^−^ molecules embedded into single-walled carbon nanotube bundles.^21^ Upon pressure increase, the *ν*(I_3_^−^) mode red-shifts linearly with pressure and subsequently disappears above 1.5 GPa due to a constricted interstitial space. Similar studies on carbon nanotubes demonstrated the linearization of PI.^22^ Further pressure increase caused disappearance of modes around 129 and 146 cm^−1^, interpreted as a decomposition of I_5_^−^ into I_3_^−^ and I_2_. The electronic structures of PI chains immobilized in a metal–organic framework and molecular sieves were probed with Raman spectroscopy as a function of pressure.^23,24^ In both cases, the confined iodine molecules formed strong donor–acceptor interactions upon compression, which was interpreted as an aggregation into one-dimensional chains. Two-dimensional polymerization has been triggered in tetraethylammonium diiodine triiodide (TEAI, formally Et_4_N^+^·I_3_^−^·2I_2_) at HP.^25^ Upon compression beyond 10 GPa, electrical conductivity of the crystal has increased by nine orders of magnitude. The observed phenomenon has been ascribed to formation of the more covalent bonds between the PI building blocks: I_2_ and I_3_^−^.

In this study, we evaluate the utility of Raman spectroscopy for the analysis of structure and composition of PI chains in TEAI at HP, up to 12 GPa. We correlate the observed spectral features with the pressure-induced evolution of the PIs molecular structure, determined previously by X-ray diffraction (XRD) experiments (Fig. 1). The features of the superficial and laser-induced iodine loss, and the role of pressure in preventing PIs decomposition are investigated. Furthermore, computational analyses are conducted on the Raman features of I_3_^−^ and I_5_^−^ systems with diverse geometries, aiming to establish a foundational guide for the preliminary identification of their structures based on spectroscopic data.

## Experimental

### Synthetic procedures

TEAI has been synthesized similarly to literature-reported protocols. Sample of 15 mmol (3.8213 g) of iodine (99.99%, Sigma-Aldrich) was dissolved in 35 ml of ethanol and heated under reflux at 40 °C till all iodine dissolved. Subsequently, 15 ml of ethanolic suspension of 3 mmol (0.7715 g) of Et_4_NI (99.9%, Sigma-Aldrich) was added dropwise. The mixture was kept under reflux at 80 °C for 3 hours, and then left to cool down overnight. Crystals were filtered off on a sintered glass, rinsed with 2 ml of cold ethanol and vacuum dried for 2 hours at room temperature. The iodine content in the sample (calc. 87.2%, exp. 86.9%) was determined by a titration with 0.010 mol dm^−3^ Na_2_S_2_O_3_ solution in the 1 : 1 mixture of diethyl ether and glacial acetic acid. The crystal structures of TEAI at HP have been determined by means of synchrotron XRD techniques, and previously reported.^25^

### Spectroscopic studies

UV-vis diffuse reflectance spectrum was recorded with a Jasco V-660 spectrophotometer (JASCO, Ishikawa-machi Hachioji-Shi Tokyo Japan), in a 200–800 nm spectral range, using BaSO_4_ as a 100% reflectance standard. Raman spectra were recorded with a HR800 (Jobin Yvon-Horiba, France) spectrometer with a spectral resolution of 1.5 cm^−1^. Spectra were collected in the reflection mode, by excitation with a 532 nm laser of a nominal power of 12 mW. The exposure time was set to 10 seconds. The laser spot size was about 4 × 4 μm. A single crystal of TEAI (150 × 80 × 20 μm) was loaded in a gas-membrane diamond anvil cell, equipped with 0.5 mm diamond (IIa class) culets. A circular sample chamber (∅ 250 μm) was drilled in a 80 μm thick stainless-steel gasket. Daphne oil 7575 (DO) was used as a pressure-transmitting medium. Pressure was calibrated using the ruby fluorescence method. Fluorescence signal was collected by the same spectrometer, by moving the laser beam onto a ruby sphere placed on a periphery of the pressure chamber. After the collection of a ruby spectrum, the laser beam was driven back onto the sample for the spectrum acquisition, with the help of a motorized XY microscope stage.

### Quantum-mechanical calculations

Quantum-mechanical calculations were performed for selected systems of I_2_–I^−^ (iodine and iodide ion) and I_2_–I_3_^−^ (iodine and triiodide ion) using Density Functional Theory (DFT). These calculations aimed to determine both the energy and Raman spectra for each system. The computational work was carried out using the Gaussian09 software,^26^ revision E.01, employing the def2tzvpp basis set and the pbe1pbe density functional.^27^ For both the I_2_–I^−^ and I_2_–I_3_^−^ systems, the geometrical configurations were systematically varied. The angles were altered from 60 to 180 degrees at 5-degree intervals, and the intermolecular distances were adjusted from 2.5 Å to 4.5 Å in 0.1 Å increments. In the I_2_–I^−^ system, this adjustment involved the angle between the I_2_ molecule and the I^−^ ion, while in the I_2_–I_3_^−^ system, it referred to the angle between the I_2_ molecule and one iodine atom in the I_3_^−^ ion. In addition, for the I_2_–I^−^ system, more detailed calculations were performed with increased resolution of the specified geometric parameters. For the range of angles from 150 to 180 degrees, the angle was varied in 1-degree increments, and the intermolecular distance for these angles was modified in finer steps of 0.05 Å. This enhanced resolution allowed for a more precise analysis of the system's behavior under these specific conditions. Calculation of Raman spectra were performed for each configuration. The specified angle and the selected distance were maintained constant throughout the calculations, and the positions of all other atoms in the systems being optimized.

## Results and discussion

### Spectroscopic features at ambient pressure

**Fig. 2:**
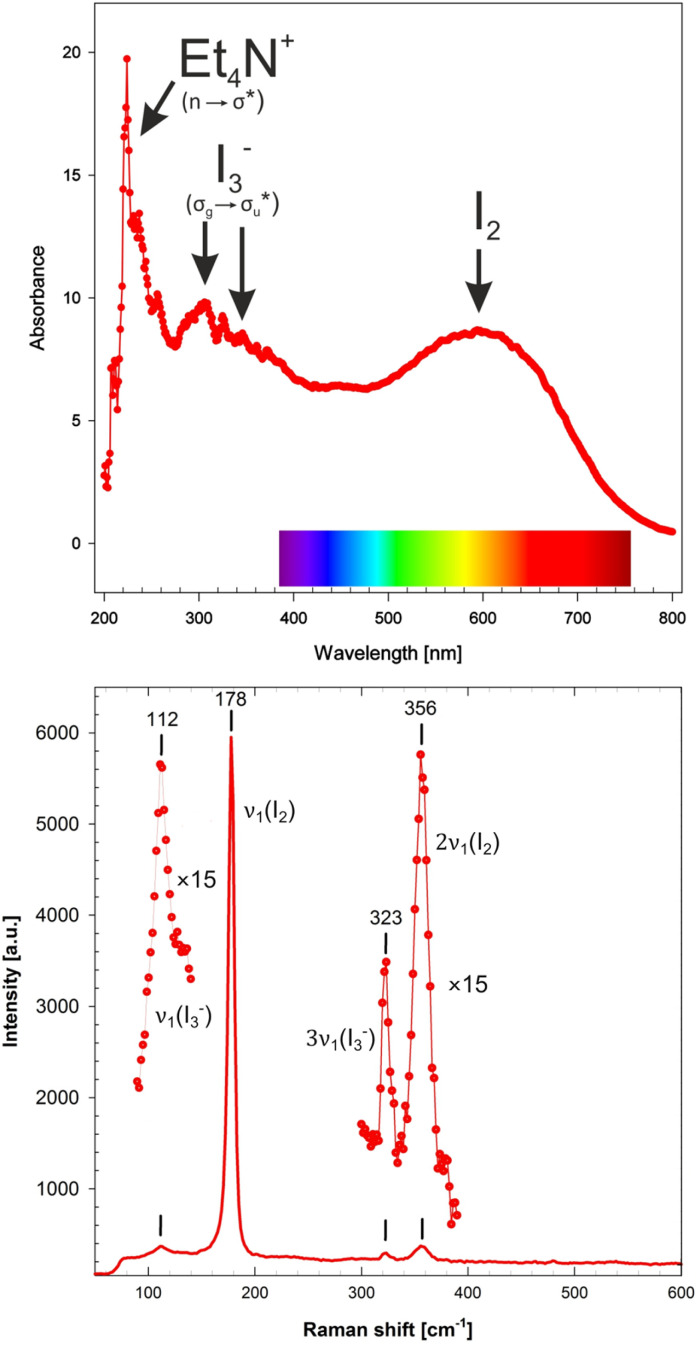
UV-vis (top) and Raman (bottom) spectrum of solid TEAI at ambient pressure.

To determine the appropriate laser excitation for the resonant enhancement of a Raman signal, diffuse reflectance spectroscopy measurements on solid TEAI sample in UV-vis range were carried out. The collected spectrum (Fig. 2 top) presents three distinct maxima: one around 230 nm due to excitation of the organic cations, mixed and overlapping maxima at 290 and 340 nm which are attributed to spin–orbit coupling in triiodides, and one broad band centered at 590 nm due to the absorption of I_2_ molecules in the visible region. According to the spectral image, the most resonating available laser wavelength was chosen: 532 nm (Nd:YAG laser, *vide* inset Fig. 2 top).

Raman spectrum of TEAI inside a sealed, but not pressurized (<0.03 GPa) DAC filled with DO was collected in a range of 50 to 600 cm^−1^ (Fig. 2 bottom). Neither Raman bands from diamonds nor from the pressure medium affect the data collected in this range. Strong diamond peak appears around 1330 cm^−1^, and bands from tertiary alkylsilanes (main constituents of Daphne oils) emerge above 600 cm^−1^.

Obtained results generally correspond to those reported for Et_4_NI_7_ in the literature. The strong band at 178 cm^−1^ corresponds to *ν*_1_(I_2_), shifted towards lower frequencies, compared to I_2_ in the solid state (180 cm^−1^). The observed shift is due to the elongation of the I–I bond engaged in the donor–acceptor interaction with I_3_^−^. Asymmetric peak corresponding to *ν*_1_(I_2_) at 178 cm^−1^ in the collected spectrum can be deconvoluted into two *ν*_1_(I_2_) bands centered at 178 and 171 cm^−1^, respectively (Fig. S1†). This bifurcation, indicative of non-symmetrical charge-transfer interactions, has been previously identified in FT-Raman spectra of TEAI (around 167 cm^−1^)^8,9^ and noted in other heptaiodides.^28^ The intensity of the peak is greatly enhanced with respect to some other studies by the resonance with the exciting radiation (532 nm).^8,9^ The single peak at 112 cm^−1^ refers to the symmetric stretching of *ν*_1_(I_3_^−^) (Tables S1 and S2†). Thus, the PI system can be seen as: (I_2_–I_3_^−^–I_2_)_*n*_. This description corresponds to the reported TEAI molecular structure.^25,29^ The iodine motif is composed of I_3_^−^ (I2–I1–I2, Fig. 1) forming two asymmetric donor–acceptor I_3_^−^⋯I_2_ interactions at ambient pressure: 3.479(4) and 3.494(4) Å (I2⋯I4 and I1⋯I3, Fig. 1), respectively, in a perpendicular fashion. A closer inspection of the Raman spectrum identifies two other bands. One at 356 cm^−1^ corresponds to overtone 2*ν*_1_(I_2_), while the second, weak one at 323 cm^−1^ is attributed to 3*ν*_1_(I_3_^−^).^13^ However, we note that its intensity is comparable with the one of *ν*_1_(I_3_^−^) (Fig. 2).

### High-pressure Raman spectroscopy

Raman spectra of the TEAI crystal were collected up to 12 GPa. Additionally, the crystal surface facing the laser beam was, prior to loading in DAC, scratched with a sharpened tungsten needle, to expose inner layers in the crystal not affected by any surface effects, such as: oxidation, iodine loss or impurities from the sample environment. Raman spectra have been collected from both sites, respectively.

**Fig. 3:**
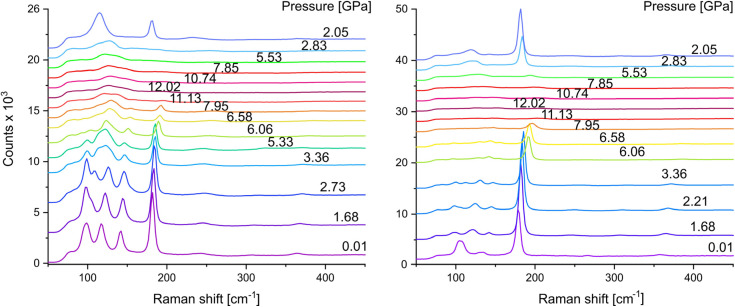
HP Raman spectra of TEAI collected at the crystal face (left) and in the ditch scratched on a crystal face (right). Displacement of the pressure labels schematically shows the compression–decompression ramp during the experiment. Estimated uncertainty of pressure determination is 0.03 GPa.

At a pressure of 0.10(3) GPa, the Raman spectra derived from these two distinct regions (the outer surface and the newly revealed internal material) exhibited fundamental variances, as depicted in Fig. 3. The Raman spectrum obtained from the freshly exposed material of the TEAI crystal closely aligns with the spectrum of pure TEAI under ambient conditions, whereas the spectrum acquired from the surface reveals a more intricate pattern, as illustrated in Fig. 3 (left panel). This raises questions regarding the origin of the additional bands observed in the superficial measurement: are they a result of decomposition, alterations in bonding induced by pressure, or a combination of these factors? In a recently published article on the iodine loss in PIs, it was observed that different spots on a surface of thiazoloquinolinium PI crystals, probed with a laser beam, resulted in different Raman spectra.^30^ It was noted that the surface of the crystal contains brown iodine-depleted patches, which instead of octaiodides (I_3_^−^–I_2_–I_3_^−^), contained only I_3_^−^. TEAI, on the contrary to the abovementioned crystals, presented a shiny monolithic surface without any visible signs of decomposition (Fig. S2†).

The first possible explanation for the additional peaks at 99 and 142 cm^−1^, observed in the spectra collected on the exposed crystal surface (Fig. 3 left), could be a loss of *D*_∞h_ symmetry of I_3_^−^. Apart from the stretching mode *ν*_1_(I_3_^−^), there are two other modes which can become Raman-active upon pressure-induced asymmetrization of triiodide: antisymmetric stretching *ν*_3_ and bending *ν*_2_, found near 140 and 70–50 cm^−1^ range, respectively.^7^ However, the structural data on the bulk crystal does not support the latter – triiodides in TEAI were shown to remain symmetrical up to 12.80(3) GPa.^25^

One can ascribe the rigidness of the band at 99 cm^−1^ during the pressure ramp (Fig. 3 left) to the intramolecular *ν*_1_(I_3_^−^) of the symmetric (*D*_∞h_) molecules. This mode may arise from the traces of the orthorhombic *Cmca* polymorph of Et_4_NI_3_ (decomposition product of TEAI containing symmetrical triiodide fragments), as the intramolecular distances in non-interacting I_3_^−^ are affected by pressure to much less an extent.^31^

**Fig. 4:**
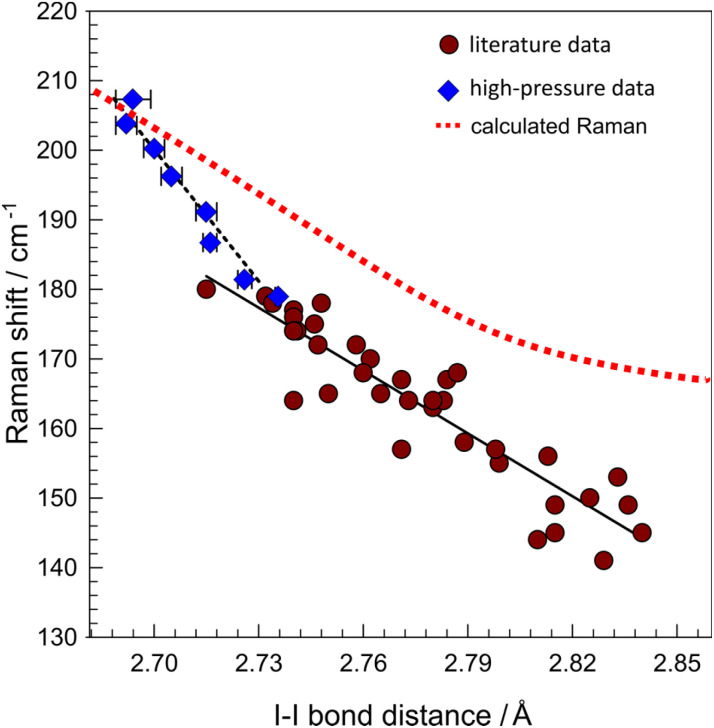
Correlations of the Raman shifts, and I–I bond lengths in high-pressure (diamonds) and ambient-pressure literature-derived (circles) data for TEAI. Black solid and dashed lines represent linear fits for two data sets (*R*^2^ = 0.968 and 0.855), respectively.

At 1.68 GPa, an additional mode at 109 cm^−1^ emerges, seen as a split of the 99 cm^−1^ peak. This band reproduces well the position and pressure-shift rate of B_3g_ in-phase internal-stretching mode of I_2_, as found by HP Raman on the iodine single crystal.^32^ The remaining two bands: 123 (*ν*_1_) and 144 cm^−1^ (*ν*_3_) [both at 1.68 GPa] can be assigned to the asymmetric (*C*_∞v_) I_3_^−^ fragments in the orthorhombic *Pnma* polymorph of Et_4_NI_3_ (two symmetry-independent units).^31^ Overall, the crystals of TEAI left in air likely seem to passivate with the layer of mixture of Et_3_NI_3_ polymorphs due to superficial decomposition:
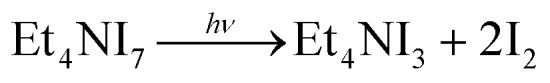
which may prevent further iodine loss from the bulk. Noteworthily, the Raman spectrum measured from the crystal in air (at ambient temperature, 15 mW laser power) yielded spectrum typical for symmetric (*D*_∞h_) I_3_^−^ fragments, (*ν*_1_(*σ*_g_) = 109 cm^−1^, Fig. S3†) due to immediate decomposition into Et_4_NI_3_. Pressure-evolution of the peak at 182 cm^−1^ (at 0.01 GPa), present on the spectra from both spots on the crystal, and attributed to *ν*_1_(I_2_), shows similar trend. In the applied pressure range, the *ν*_1_(I_2_) shift linearly: 2.12(8) and 2.17(7) cm^−1^ GPa^−1^, for the crystal surface and freshly exposed material, respectively (Fig. S4†), and its slope was used to correlate with the I–I interatomic distances derived from HP XRD experiments and Raman shifts (Fig. 4).

As the intramolecular I–I distance compresses, the Raman spectrum shows a blue shift (Fig. 3 and 4). Influence of HP on the shift magnitude is more pronounced (−630(50) cm^−1^ Å^−1^, Fig. 4) than for “chemical pressure” exerted by the supramolecular environment in the literature-derived dataset (−300(20) cm^−1^ Å^−1^, the dataset includes only structures containing I_2_⋯I_3_^−^ contacts in almost perpendicular arrangements, see Table S3† for details). It is because pressure delivers high mechanical energy to the system through compression, forcing the structure to explore the otherwise energetically unfavorable regions on a potential energy surface. Literature data gathered for ambient-pressure structures (*n* = 44) fits rather poorly with a linear regression model (*R*^2^ = 0.855, Fig. 4). Among the reasons for this disparity are: (1) high uncertainties in Raman shift determination (up to 4 cm^−1^); (2) inconsistent temperatures between XRD data (large range of temperatures) and Raman data (mostly collected at room temperature); (3) influence of the crystal field. Therefore, it is difficult to precisely estimate the interatomic distance in PIs, based solely on the Raman shifts of the modes, and using the correlation made with ambient pressure structures with various geometries.

**Fig. 5:**
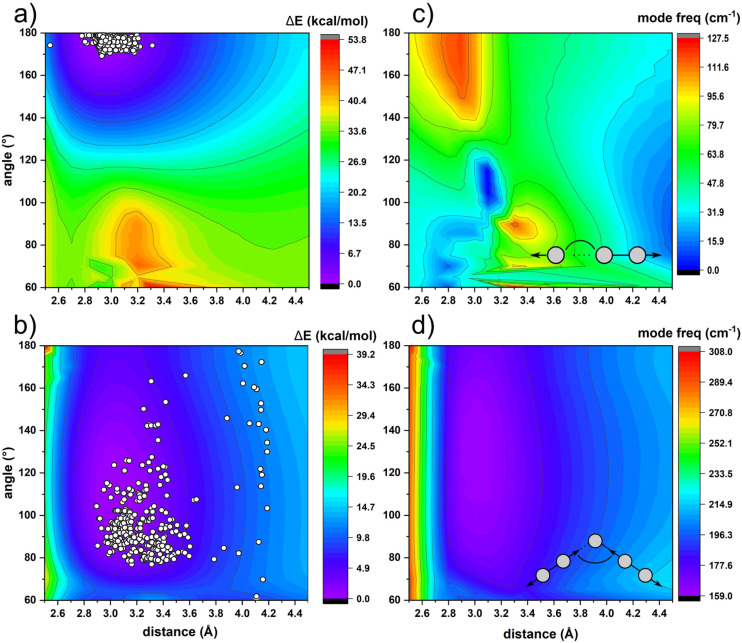
Potential energy surfaces calculated for tri- and pentaiodide fragments with different geometries are shown in (a) and (b), respectively. The white circles represent structures derived from CSD. The calculated Raman shifts, for the corresponding geometries, of I–I symmetric stretch for triiodide (I_2_⋯I^−^) and pentaiodide (I_2_⋯I_3_^−^) are shown in (c) and (d), respectively.

An important difference in a compression mode between the two data sets (Fig. 4) is the role and behavior of intermolecular interactions (I2⋯I4 and I1⋯I3, Fig. 1). In general, as the electrophilic I_2_ approaches an electron-donor (*e.g.* I_3_^−^), the stretching *ν*_1_(I_2_) shifts towards lower frequencies with respect to solid iodine (180 cm^−1^), due to the intramolecular distance elongation.^33^ In other words, the shorter intermolecular I⋯I distance, the longer intramolecular I–I distance, due to higher occupation of σ* molecular orbital. In case of HP structures, even though I_2_ moves closer to the donor I_3_^−^, the frequency increases slightly due to the pressure-induced intramolecular bond shortening, which compensates for the bond elongation caused by the higher occupation of σ* molecular orbital. Most of the reported structures of triiodides consists of linear and nearly symmetrical species (Fig. 5a). The energy needed to deform I_3_^−^ changes continuously as the distance between its building blocks, I_2_ and I^−^, increases (Fig. S5†). The corresponding Raman spectra reflect the characteristics of the potential energy surface. The frequency of *ν*_1_(I_3_^−^) decreases upon deformation from the most favourable symmetric case (*d*_I–I_ ∼ 2.933 Å, Fig. 5c). Concomitantly, *ν*_2_(I_3_^−^) frequency increase has much steeper slope upon I–I bond compression than expansion (Fig. S6†). It reflects the situation observed in this study and explains the slope difference shown in Fig. 4. Bond deformation (*d*_I⋯I_ > 3.6 Å) leads eventually to asymmetrization of I_3_^−^ into I_2_ and I^−^ and emergence of the *ν*_I–I_ Raman band around 180 cm^−1^, typical for internal stretching in solid iodine.^34^ At the larger separation *ν*_I–I_ approaches 203 cm^−1^ – a value in between liquid (194 cm^−1^) and gaseous (213 cm^−1^) iodine.^35^ Analysis of *ν*_2_/*ν*_1_ modes intensity ratio can additionally be used to estimate the degree of moderate asymmetry of triiodides, showing a sudden increase of *ν*_2_/*ν*_1_ intensity ratio above *d*_I⋯I_ = 3.2 Å (Fig. S7†). Discontinuous character of this change might serve as a benchmark distance differentiating I_3_^−^ units and I_2_·I^−^ assembly. In case of pentaiodide fragments, which are present in TEAI, the deformation energy is much lower than in triiodides, and allows for a high structural flexibility in solid state. It is reflected by a broad distribution of the observed geometries in CSD (Fig. 5b). Changes in the symmetric I–I stretching Raman modes somehow reflect this situation. Unlike in I_3_^−^, compression of the inner I–I distance below the distance typical for solid I_2_ (∼2.72 Å) causes a rapid increase in frequency of this mode (Fig. 5d). Such a level of compression has not yet been observed in both ambient and high-pressure structures of polyiodides.

However, separation of I_5_^−^ into, as well as changes in the dihedral angle (*α*_I–I_) between them, has a minor effect on the observed frequencies (Fig. 5d). Therefore, it is difficult to estimate the geometry of higher PIs solely by the analysis of mode frequencies on the Raman spectra. The intensity ratio between the symmetric (*ν*_1_) and asymmetric (*ν*_2_) outer stretching bands in I_5_^−^ (166 and 144 cm^−1^ in the optimized structure, respectively) is much less indicative than in I_3_^−^ (Fig. S8†) and is strongly laser-frequency dependent. For example, in (CH_3_)_4_NI_5_ (*d*_I⋯I_ = 3.14 Å, *α*_I–I_ = 95°) the intensity ratio changes from ∼0.8 at 1064 nm to 1.0 at 568.2 nm incidence wavelength, due to resonance Raman effect.^8^

Noteworthily, the structural changes are continuous in both probed sites of the TEAI crystal at HP, even beyond the hydrostatic limit of DO (*circa* 4.0 GPa (ref. 36)). Above 4.0 GPa, the Raman bands broaden, and the intensity drops. At 11.13 GPa, the most intense *ν*_1_(I_2_) cannot be discerned anymore from the background. Although the intensity loss and bandwidth broadening are the common features in HP Raman, the observed intensity loss might be due to the formation of multiple short I–I interactions in a highly compressed structure. In fact, electrical resistivity measurements indicated that above 10 GPa TEAI undergoes an insulator–semiconductor transition, which is coupled with a transformation into a PI polymeric system. Upon pressure release, some of the Raman bands reappear. The freshly exposed TEAI in the crystal interior recovers nearly identical as at the similar pressure at the beginning of the experiment, with the comparable *ν*_1_(I_3_^−^) : *ν*_1_(I_2_) ratio. In case of the crystal surface, there is an increase of *ν*_1_(I_3_^−^) : *ν*_1_(I_2_) intensity ratio. Features of the Raman spectrum at this point closely resemble those reported for a mixture of TEAI and Et_4_NI_3_, as discussed above. One possible explanation why decomposition can be detected only for the spectra collected from the surface is that a crystal moved during the rapid decompression. As a result, the laser could probe a different spot with different ratio of PIs. On the other hand, the probing laser spot size is much smaller than the width of the ditch containing the fresh material, the small crystal movement does not affect the observed spectral features.

### Pressure-stabilization of donor–acceptor polyiodide chains

**Fig. 6:**
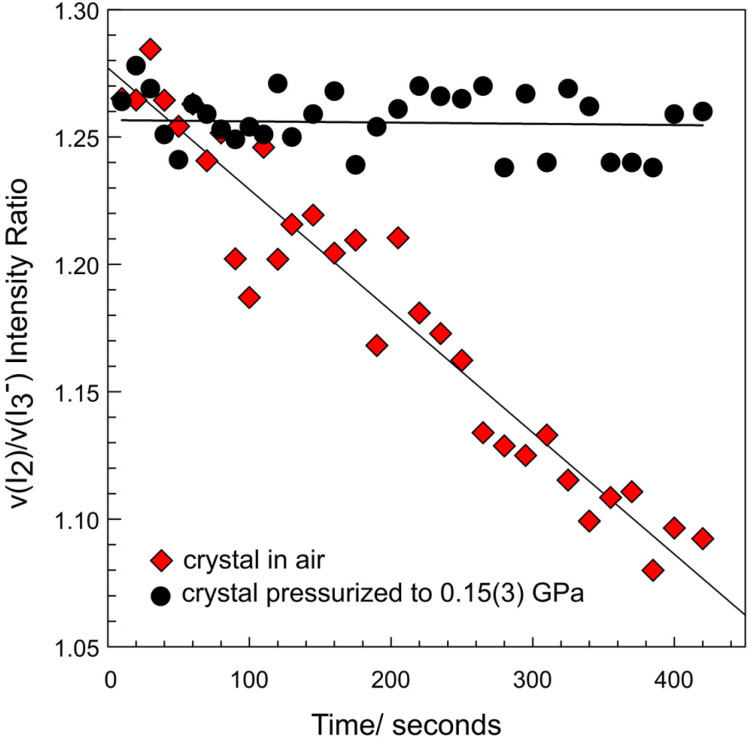
Linear decay of *ν*_1_(I_2_) and emergence of *ν*_1_(I_3_^−^) in TEAI upon continuous 532 nm laser irradiation (10 mW) kept in air (diamond), and in closed DAC at 0.15(3) GPa (circles), respectively. Solid lines represent corresponding linear fits.

Two single-crystals of TEAI, one in air and one placed in DAC filled with DO pressurized to 0.15(3) GPa, respectively, have been irradiated continuously with a 10 mW 532 nm laser. The Raman data has been collected in 10-second intervals for an overall 420 seconds (Fig. 6). Intensity of *ν*_1_(I_2_) in the crystal kept in air was found to diminish linearly as the signal of *ν*_1_(I_3_^−^) emerges (Fig. 3 and 6) upon irradiation. Laser light excites weakly-bonded I_2_ molecules, fostering their sublimation according to the reaction scheme shown above. As an effect, tetraethylammonium triiodide is formed. Increase of the laser power to 25 mW causes the crystal to sublimate instantaneously, leaving gaps in the crystal (see ESI Video†). Similar laser-sensitivity has been observed in Cs_2_I_8_, where an increase of the laser power caused gradual formation of triiodides with different geometries, and ultimately decomposition to CsI.^12^ It has been suggested that the laser photolysis process yields atomic iodine. In solutions, such atomic species can be scavenged by I_3_^−^, and lead to formation of higher PIs.^37^ In the case of pressurized TEAI, the triiodide fragments are already at the short intermolecular distances to iodine molecules. And the photolysis is not noticeable over the studied time scale (Fig. 6 and Fig. S9†). In fact, we have already noticed a pressure-aided stabilization in *N*-propylurotropinium heptaiodide (UrPrI_7_) against thermal decomposition.^16^ The stabilization mechanism, both in TEAI and UrPrI_7_, likely relies on the strengthening of the intermolecular interactions between I_2_ and I_3_^−^ fragments in the pressurized crystal, as discussed above.

## Conclusions

TEAI has been studied using HP Resonant Raman Spectroscopy up to 12 GPa. The Raman spectra show substantial differences whether the data was collected on the surface or inside freshly exposed crystal interior. The surface of TEAI is covered with the decomposition products, likely the mixture of Et_4_NI_3_ (*Cmca* and *Pnma* polymorphs) and iodine. The crystal interior shows Raman features typical for pristine TEAI. Raman spectroscopy of PI is complementary to XRD techniques, as it can differentiate between PIs in the bulk and on the surface of the crystal. Upon compression *ν*_1_(I_2_) shifts linearly with pressure, as the intramolecular I–I distances shorten continuously. The I–I bond compression rate can be correlated with a Raman shift of −630(50) cm^−1^ Å^−1^, a much higher rate than observed in case of the “chemical-pressure” effect exerted by a crystal-field in ambient-pressure structures (−300(20) cm^−1^ Å^−1^). Pressure-confinement at as low as 0.15(3) GPa significantly hinders a laser-induced decomposition of TEAI into lower PIs. The effect is particularly pronounced in the pristine TEAI.

To obtain reliable results from Raman experiments on PIs, only the freshly prepared sample should be investigated. This study shows that even in case of high-power output of the laser source, the photodecomposition of I_3_^−^⋯I_2_ chains in TEAI can be prevented. Resonance Raman spectroscopy combined with high pressure additionally allows differentiation of multiple PIs in the complex spectral image, based on the different intensity ratio and frequency pressure-shifts of certain bands. Such determination might be precluded by using only ambient-pressure samples, due to decomposition and overlap of multiple bands associated with higher PIs.

## Conflicts of interest

There are no conflicts to declare.

## Supplementary Material

DT-053-D4DT00268G-s001

DT-053-D4DT00268G-s002
