# Supplementary material for: Pressure stabilization effect on the donor–acceptor polyiodide chains in tetraethylammonium bis(diiodine) triiodide – insights from Raman spectroscopy
Source: Dalton Trans. 2024 Feb 15;53(11):5152–9. doi: 10.1039/d4dt00268g (PMC10928838; doi:10.1039/d4dt00268g)
Supplement: DT-053-D4DT00268G-s002 [file DT-053-D4DT00268G-s002.pdf]

Electronic Supplementary Material (ESI) for Dalton Transactions.
This journal is © The Royal Society of Chemistry 2024

Supplementary Material

Pressure stabilization effect on the donor-acceptor polyiodide chains in tetraethylammonium bis(diodine) triiodide – insights from Raman spectroscopy

Tomasz Poręba[a], Piero Macchi[b], Nicola Casati[c] and Tomasz Sierański[d]*

[a.] *Laboratory for Quantum Magnetism, Institute of Physics, École Polytechnique Federale de Lausanne, Lausanne CH-1015, Switzerland*
[b.] *Department of Chemistry, Materials and Chemical Engineering, Polytechnics of Milan. Via Mancinelli 7, 20131, Milan, Italy*
[c.] *Swiss Light Source, Paul Scherrer Institute. CH-5232 Villigen PSI, Switzerland*
[d.] *Institute of General and Ecological Chemistry, Lodz University of Technology, Zeromskiego 116, 90 - 924 Lodz, Poland*
**Corresponding author: tomasz.poreba@esrf.fr*

1. Raman spectroscopy details on TEAI

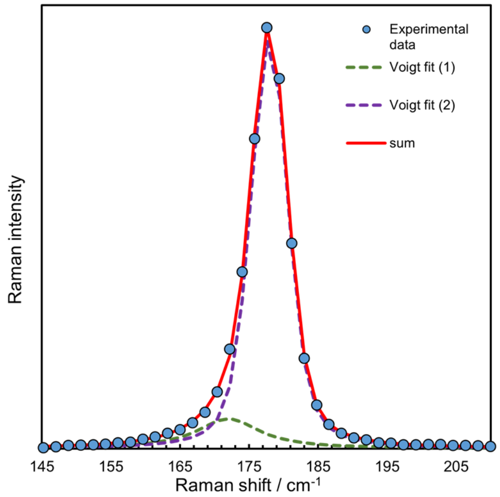

Figure S1. Experimental ν(I_2) peak profile in TEAI deconvoluted with two Voigt functions centred at 171 and 178 cm^{-1}, respectively.

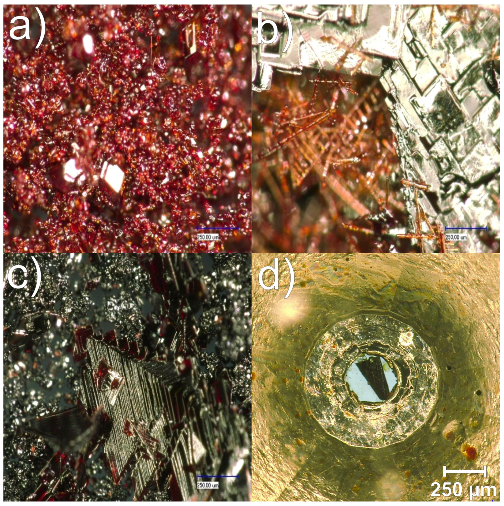

Figure S2. Sample microphotographs: a) Et_4NI_3, b) mixture of Et_4NI_3 polymorphs at TEAI, c) pristine TEAI, d) TEAI crystal loaded in DAC

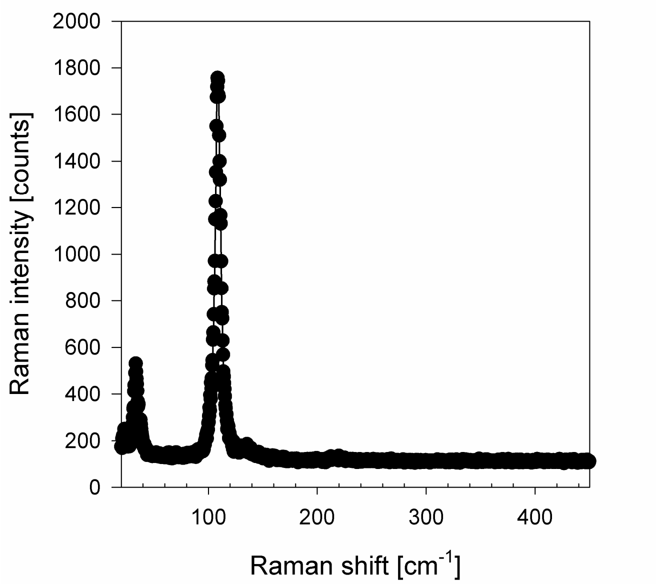

Figure S3. Raman spectrum of TEAI crystal collected in air (10mW, 10s, 532 nm) presents immediate decomposition to Et_4NI_3

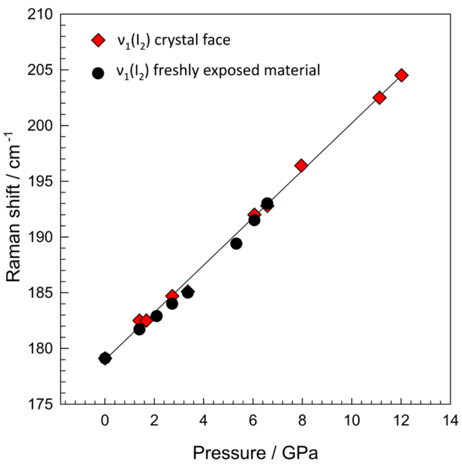

Figure S4. Pressure-shift of $\nu_1(I_2)$ mode in TEAI observed from a region on a surface (diamond) and freshly exposed crystal interior (circles)

Table S1: Bond lengths for the studied systems at their energetic minima.

System	Bondlength (Å)	Angle (°)
**I_3^-**	2.933	180.00
**I_2-I_3^-**	3.030 (inner)	109.20
	2.827 (outer)	

Table S2: Raman data (frequency and activity) for studied compounds for which an energetic minimum has been found.

System	Frequency [cm^{-1}] (activity)		
	Bending	Assym. Stretch	Symmetric Stretch
I_3^-	58.66 (0)	115.92 (46.6)	145.42 (0)
I_2-I_3^-	-	144.37 (50.2)	160.26 (149.4)

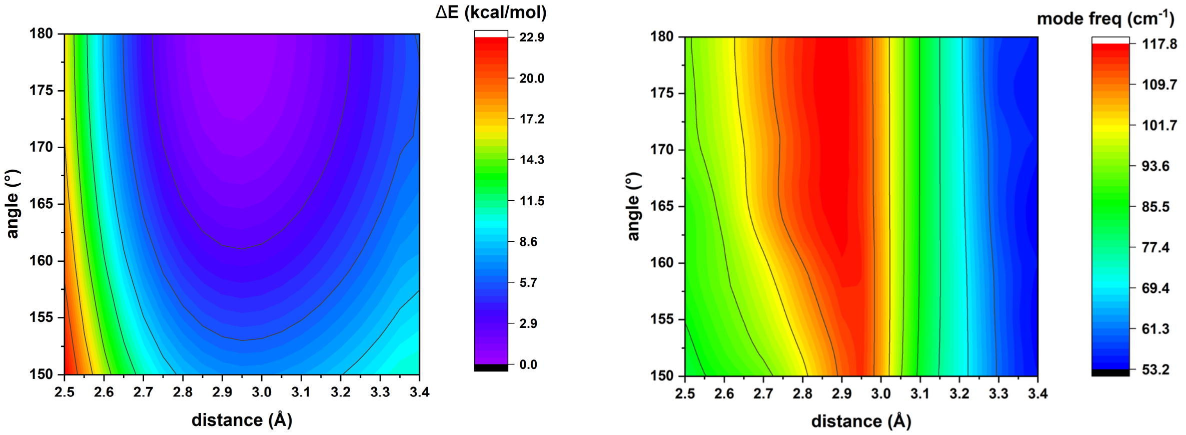

Figure S5. Potential energy surfaces calculated for triiodide fragment with different geometries are shown (left) along with the calculated Raman shifts (right), for the corresponding geometries, of I-I stretch for triiodide ($I_2 \cdots I^-$)

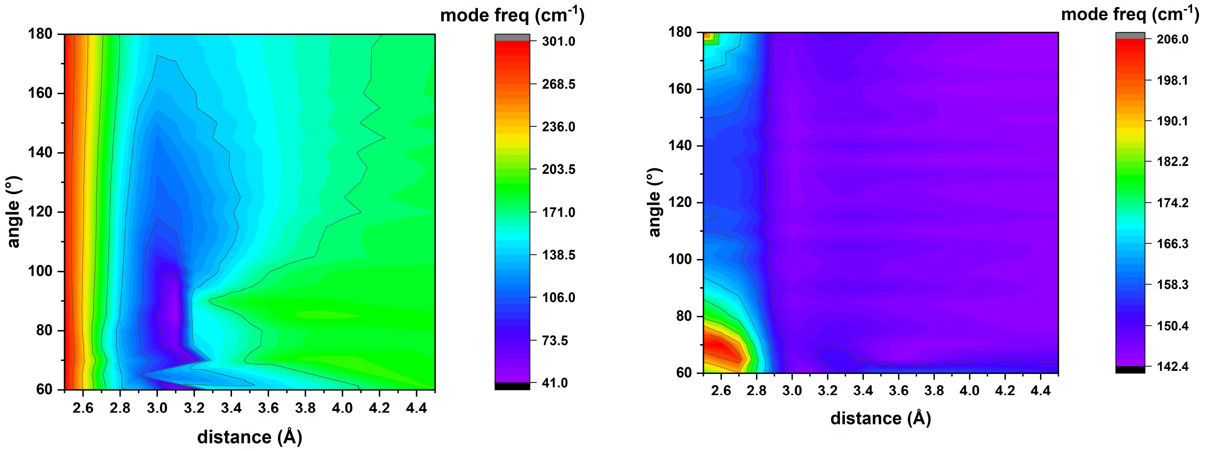

Figure S6. Asymmetric stretch, $\nu_2(I_3^-)$ (left) and $\nu_2(I_5^-)$ (right), mode frequencies calculated for various geometries

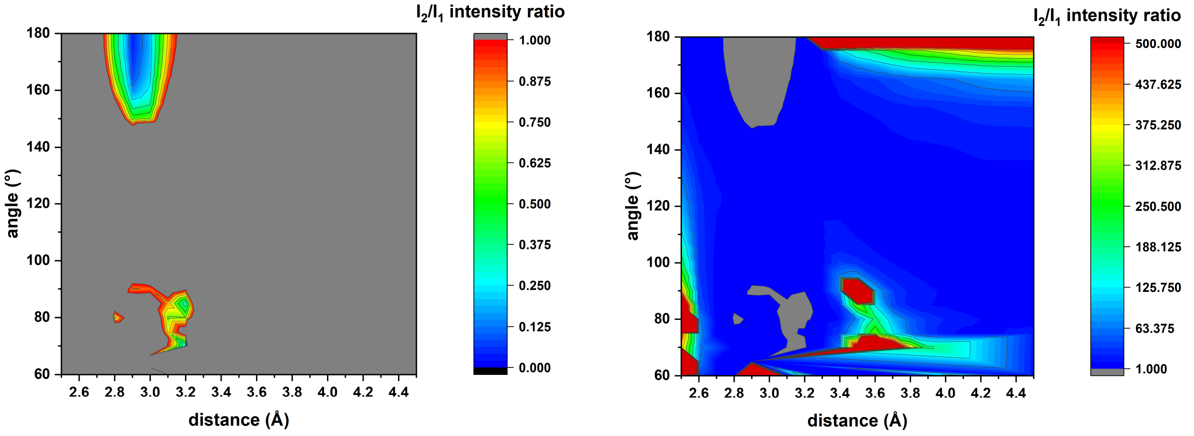

Figure S7. ν_2/ν_1 ($\sigma_{u,}$,~140 cm^{-1} and σ_g,~102 cm^{-1}) modes intensity ratios calculated for various I_3^- geometries: below (left) and above (right) 1.

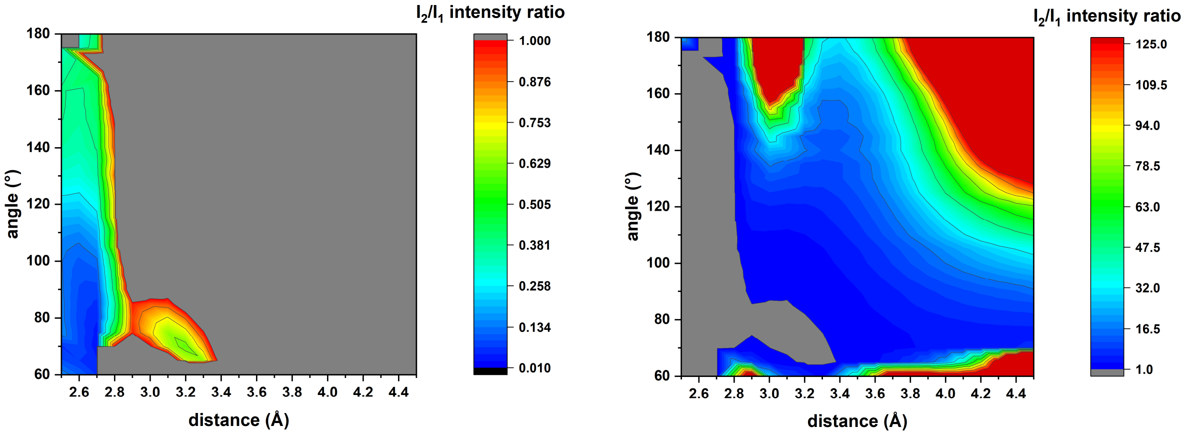

Figure S8. ν_2 (symmetric outer stretch)/ν_1 (asymmetric outer stretch) [σ_g ,~165 cm^{-1}and σ_u, ~144 cm^{-1}] modes intensity ratios calculated for various I_5^- geometries: below (left) and above (right) 1.

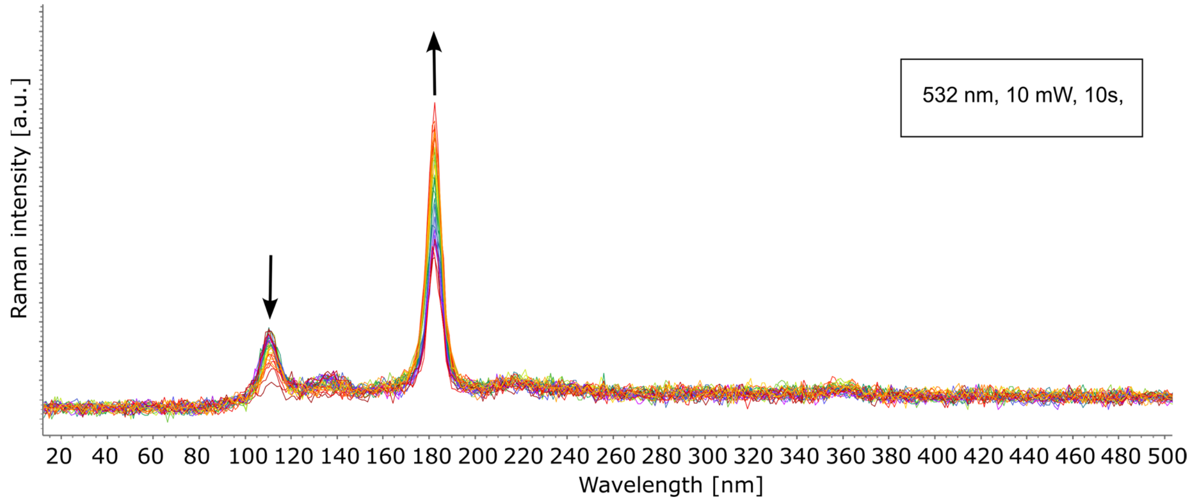

Figure S9. Linear decay of $\nu_1(I_2)$ and emergence of $\nu_1(I_3^-)$ in TEAI upon continuous 532 nm laser irradiation kept in air.

2. Experimental structures containing donor-acceptor polyiodides

Table S3. Experimental I-I distances and the respective Raman shifts extracted from literature. Only structures containing V - or L-shaped $I_2 \cdot I_3^-$ fragments were considered.

Compound	I-I bond length in I_2 [Å]	Raman shift [cm^{-1}]	References
**nicotine-1,1′-diium bis(triiodide)-diiodine**	2.740	177	[1]
**1-(Iodomethyl)-1,2-dihydro[1,3]thiazolo[3,2-a]quinolin-10-ium tri-iodide hemikis(di-iodine)**	2.765	165	[2]
**solid iodine**	2.715	180	[3,4]
**(Z)-4-chloro-5-((2-((4-chloro-5*H*-1,2,3-dithiazol-5-ylidene)amino)phenyl)amino)-1,2,3-dithiazol-1-ium oligoiodide**	2.732	179	[5]
**(E)-3-(iodomethylene)-2,3-dihydro-[1,4]oxazino[2,3,4-ij]quinolin-4-ium triiodide diiodine**	2.747	172	[6]
**4-benzoyloxy-2,2,6,6-tetramethylpiperidinyl-1-oxy tetrakis(diiodine) di(pentaiodide)**	2.829	141	[7]
**4-benzoyloxy-2,2,6,6-tetramethylpiperidinyl-1-oxy tetrakis(diiodine) di(pentaiodide)**	2.813	156	[7]
**5,10,15,20-tetraphenylprophyrinium bis(pentaiodide)**	2.740	174	[8]
**tetraethylammonium bis(diiodine) triiodide**	2.734	178	[9,10]
**tetramethylammonium pentaiodide**	2.799	155	[10,11]
**N-Propylurotropinium heptaiodide**	2.740	176	[12,13]
**Tetra(n-propyl)ammonium bis(iodine) triiodide**	2.740	164	[14,15]
**N-propylurotropinium pentaiodide**	2.780	163	[13,16]
**N-propylurotropinium pentaiodide**	2.815	149	[13,16]
**bis(N-methylurotropinium) octaiodide**	2.773	164	[13,17]
**tris(morpholinecarbodithioato-S,S′)manganese(IV)pentaiodide–dichloromethane (2/1)**	2.810	144	[18]
**tris(morpholinecarbodithioato-S,S′)manganese(IV)pentaiodide–dichloromethane (2/1)**	2.750	165	[18]
**morpholinium pentaiodide**	2.783	164	[19,20]
**bis(3,5-Di-(N-morpholinio)-1,2,4-trithiolane) hexadecaiodide**	2.741	174	[20,21]
**bis(phenacetinium) pentaiodide**	2.748	178	[22,23]
**bis(phthalocyaninato)niobium(IV) octaiodide, 1-chloronaphtalene solvate (3.5/1)**	2.771	167	[24]
**bis(N-methylbenzothiazole-2(3H)-selone)-µ-iodonium heptaiodide**	2.771	157	[25]
**bis(N-methylbenzothiazole-2(3H)-selone)-µ-iodonium heptaiodide**	2.746	175	[25]
**cesium octaiodide**	2.762	170	[26,27]

**(acetonitrile)-(3,6,9-trithia-1(2,9)-1,10-phenanthrolinacyclodecaphane)-nickel(II) triiodide pentaiodide**	2.784	167	[28]
**bis(1,4,7-trithiacyclononane)-cobalt(III) tris(tri-iodide) iodine**	2.787	168	[29]
**bis(1,4,7-trithiacyclononane)-cobalt(III) tris(tri-iodide) iodine**	2.825	150	[29]
**bis(1,4,7-trithiacyclononane)-nickel(II) bis(pentaiodide)**	2.780	164	[29]
**bis(1,4,7-trithiacyclononane)-nickel(II) bis(pentaiodide)**	2.815	145	[29]
**(1,4,8,11-tetrathiacyclotetradecane)-palladium(II) bis(pentaiodide) acetonitrile solvate**	2.760	168	[29]
**(1,4,8,11-tetrathiacyclotetradecane)-palladium(II) bis(pentaiodide) acetonitrile solvate**	2.840	154	[29]
**(1,4,8,11-tetrathiacyclotetradecane)-palladium(II) bis(pentaiodide) acetonitrile solvate**	2.798	163	[29]
**catena-((μ_2-Iodo)-bis((tetrathia-16-crown-4)-palladium(II)) undecaiodide)**	2.836	149	[30]
**catena-((μ_2-Iodo)-bis((tetrathia-16-crown-4)-platinum(II)) undecaiodide)**	2.789	158	[30]
**catena-((μ_2-Iodo)-bis((tetrathia-16-crown-4)-platinum(II)) undecaiodide)**	2.833	153	[30]

Bibliography

[1] G.J. Reiss, I 5 – polymers with a layered arrangement: synthesis, spectroscopy, and structure of a new polyiodide salt in the nicotine/HI/I 2 system, Zeitschrift Für Naturforsch. B. 70 (2015) 735–739. https://doi.org/10.1515/znb-2015-0092.

[2] I.D. Yushina, D.G. Pikhulya, E. V. Bartashevich, The features of iodine loss at high temperatures, J. Therm. Anal. Calorim. 139 (2020) 1017–1023. https://doi.org/10.1007/s10973-019-08442-y.

[3] A. Congeduti, M. Nardone, P. Postorino, Polarized Raman spectra of a single crystal of iodine, Chem. Phys. 256 (2000) 117–123. https://doi.org/10.1016/S0301-0104(00)00085-9.

[4] P.M. Harris, E. Mack, F.C. Blake, THE ATOMIC ARRANGEMENT IN THE CRYSTAL OF ORTHORHOMBIC IODINE, J. Am. Chem. Soc. 50 (1928) 1583–1600. https://doi.org/10.1021/ja01393a009.

[5] O.I. Bol’shakov, I.D. Yushina, E. V Bartashevich, Y. V Nelyubina, R.R. Aysin, O.A. Rakitin, Asymmetric triiodide-diiodine interactions in the crystal of (Z)-4-chloro-5-((2-((4-chloro-5H-1,2,3-dithiazol-5-ylidene)amino)phenyl)amino)-1,2,3-dithiazol-1-ium oligoiodide, Struct. Chem. 28 (2017) 1927–1934. https://doi.org/10.1007/s11224-017-0987-y.

[6] I.D. Yushina, B.A. Kolesov, E. V. Bartashevich, Raman spectroscopy study of new thia- and oxazinoquinolinium triodides, New J. Chem. 39 (2015) 6163–6170. https://doi.org/10.1039/C5NJ00497G.

[7] X. Pang, H. Wang, X.R. Zhao, W.J. Jin, Anionic 3D cage networks self-assembled by iodine and V-shaped pentaiodides using dimeric oxoammonium cations produced in situ as templates, Dalt. Trans. 42 (2013) 8788–8795. https://doi.org/10.1039/c3dt50191d.

[8] P. Deplano, F.A. Devillanova, J.R. Ferraro, F. Isaia, V. Lippolis, M.L. Mercuri, On the Use of Raman Spectroscopy in the Characterization of Iodine in Charge-Transfer Complexes, Appl. Spectrosc. 46 (1992) 1625–1629. https://doi.org/10.1366/0003702924926880.

[9] Y. Wang, Y. Xue, X. Wang, Z. Cui, L. Wang, The stable polyiodides: Experimental and theoretical studies of formation mechanism, J. Mol. Struct. 1074 (2014) 231–239. https://doi.org/10.1016/j.molstruc.2014.05.062.

[10] E.M. Nour, L.H. Chen, J. Laane, Far-infrared and Raman spectroscopic studies of polyiodides, J. Phys. Chem. 90 (1986) 2841–2846. https://doi.org/10.1021/j100404a014.

[11] M.S. Abdelbassit, O.J. Curnow, M.K. Dixon, M.R. Waterland, The Binary Iodine-Chlorine Octahalide Series [I n Cl 8− n] 2− (n =3, 3.6, 4), Chem. – A Eur. J. 25 (2019) 11650–11658. https://doi.org/10.1002/chem.201902135.

[12] K.-F. Tebbe, T. Farida, H. Stegemann, H. F�llbier, Untersuchungen an Polyhalogeniden. XXIII. Kristallstrukturen der N-Alkylurotropiniumtriiodide UrRI3 mit R = Methyl, Ethyl, n-Propyl und n-Butyl, Zeitschrift F�r Anorg. Und Allg. Chemie. 622 (1996) 525–533. https://doi.org/10.1002/zaac.19966220323.

[13] H. Mittag, H. Stegemann, H. Füllbier, G. Irmer, Raman spectroscopic investigation of N-alkylurotropinium polyiodides, J. Raman Spectrosc. 20 (1989) 251–255. https://doi.org/10.1002/jrs.1250200409.

[14] K.-F. Tebbe, T. Gilles, Untersuchungen an Polyhalogeniden. XXVII. �ber Tetra(n-propyl)ammoniumpolyiodide (n-Pr4N)In mit n = 3, 5, 7: Darstellung und strukturelle Charakterisierung eines Triiodids (n-Pr4N)I3, eines Pentaiodids (n-Pr4N)I5 und eines Heptaiodids (n-Pr4N)I7, Zeitschrift F�r Anorg. Und Allg. Chemie. 622 (1996) 1587–1593. https://doi.org/10.1002/zaac.19966220923.

[15] F.W. Parrett, N.J. Taylor, Spectroscopic studies on some polyhalide ions, J. Inorg. Nucl. Chem. 32 (1970) 2458–2461. https://doi.org/10.1016/0022-1902(70)80541-3.

[16] K.-F. Tebbe, K. Nagel, Untersuchungen an Polyhalogeniden. XXVI [1]. �ber N-Propylurotropiniumpolyiodide UrPrIx mit x = 5 und 7: Strukturelle Charakterisierung eines Pentaiodids und eines Heptaiodids, Zeitschrift F�r Anorg. Und Allg. Chemie. 622 (1996) 1323–1328. https://doi.org/10.1002/zaac.19966220809.

[17] A. Gräfe-Kavoosian, S. Nafepour, K. Nagel, K.-F. Tebbe, On the Octaiodide Ion I82-: Preparation and Crystal Structure of [(Crypt-2.2.2)H2]I8, of [Ni(phen)3]I8-2CHCl3 and of the Bis(Nalkylurotropinium)octaiodides (UrR)2I8 with R = methyl and ethyl, Z. Naturforsch. 53b (1998) 641–652.

[18] P. Deplano, E.F. Trogu, F. Bigoli, M.A. Pellinghelli, Synthesis and crystal structure of tris(morpholinecarbodithioato-S,S′)manganese(<scp>IV</scp>)pentaiodide–dichloromethane (2/1), J. Chem. Soc., Dalt. Trans. (1987) 2407–2410. https://doi.org/10.1039/DT9870002407.

[19] CSD entry: WAHZIO, (n.d.).

[20] P. Deplano, F.A. Devillanova, J.R. Ferraro, M.L. Mercuri, V. Lippolis, E.F. Trogu, FT-Raman study on charge-transfer polyiodide complexes and comparison with resonance Raman results, Appl. Spectrosc. 48 (1994) 1236–1241. https://doi.org/10.1366/0003702944027372.

[21] F. Bigoli, M.A. Pellinghelli, G. Crisponi, P. Deplano, E.F. Trogu, Reaction of bis(morpholinothiocarbonyl) disulphide with iodine. Existence of a 1 : 1 charge-transfer precursory adduct in an oxidation reaction. Isolation and crystal structure of bis[3,5-di(N-morpholinio)-1,2,4-trithiolane] hexadecaiodide, J. Chem. Soc. Dalt. Trans. (1985) 1349. https://doi.org/10.1039/dt9850001349.

[22] F.H. Herbstein, M. Kapon, The crystal structures of the polyiodide salts (phenacetin) 2 HI 5 and (theobromine) 2 H 2 I 8, Philos. Trans. R. Soc. London. Ser. A, Math. Phys. Sci. 291 (1979) 199–218. https://doi.org/10.1098/rsta.1979.0021.

[23] P. Deplano, J.R. Ferraro, M.L. Mercuri, E.F. Trogu, Structural and Raman spectroscopic studies as complementary tools in elucidating the nature of the bonding in polyiodides and in donor-I2 adducts, Coord. Chem. Rev. 188 (1999) 71–95. https://doi.org/10.1016/S0010-8545(98)00238-0.

[24] M.P. Donzello, C. Ercolani, A. Chiesi-Villa, C. Rizzoli, One Step Forward to “Stapled” Bis(phthalocyanine) Metal Complexes: Synthesis, Characterization, and Redox Properties of Bis(phthalocyaninato)niobium(IV). X-ray Crystal Structure of the Monoelectronically Oxidized Species [Pc 2 Nb](I 3)(I 2) 0.5 (ClNP) 3, Inorg. Chem. 37 (1998) 1347–1351. https://doi.org/10.1021/ic970841f.

[25] F. Demartin, P. Deplano, F.A. Devillanova, F. Isaia, V. Lippolis, G. Verani, Conductivity, FT-Raman spectra, and x-ray crystal structures of two novel [D2I]In (n = 3 and D = N-methylbenzothiazole-2(3H)-selone; n = 7 and D = N-methylbenzothiazole-2(3H)-thione) iodonium salts. First example of I-.3I2 heptaiodide, Inorg. Chem. 32 (1993) 3694–3699. https://doi.org/10.1021/ic00069a025.

[26] K.I. K Odagi, H Nakayama, Behavior of I2 and I3− Molecular Units Near the Crystal Surface of CsI and Related Polyiodides. Raman-Spectroscopic Studies, Bull. Chem. Soc. Jpn. 63 (1990) 3277–3281.

[27] E.E. Havinga, K.H. Boswijk, E.H. Wiebenga, The crystal structure of Cs2I8(CsI4), Acta Crystallogr. 7 (1954) 487–490. https://doi.org/10.1107/S0365110X54001545.

[28] M. Carla Aragoni, M. Arca, F. Demartin, F.A. Devillanova, A. Garau, F. Isaia, V. Lippolis, S. Rizzato, G. Verani, [Ni(L)(MeCN)]2+complex cation as a template for the assembly of extended I3−·I5− and I5−·I7− polyiodide networks {L=2,5,8-trithia[9](2,9)-1,10-phenanthrolinophane}. Synthesis and structures of [Ni(L)(MeCN)]I8 and [Ni(L)(MeCN)]I12, Inorganica Chim. Acta. 357 (2004) 3803–3809. https://doi.org/10.1016/j.ica.2004.05.027.

[29] A.J. Blake, W.S. Li, V. Lippolis, S. Parsons, M. Schröder, Extended structures of polyiodide salts of transition metal macrocyclic complexes, Acta Crystallogr. Sect. B Struct. Sci. (2007). https://doi.org/10.1107/S0108768106041668.

[30] A.J. Blake, R.O. Gould, W. Li, V. Lippolis, S. Parsons, M. Schröder, Thioether crown complexes as templates for the assembly of extended polyiodide networks: synthesis and structures of {[M([16]aneS4)]2I}I11 (M = Pd, Pt), [Pd(cis-HO)2[14]aneS4)](I3)2, [Pd([9]aneS3)2](Ix)2 (x = 3, 5), Cryst. Eng. 2 (1999) 153–170. https://doi.org/10.1016/S1463-0184(99)00016-7.
